# Electrochemical Impedance Spectroscopy as a Tool To Investigate the Electrochemical Behavior of Beetroot and Spinach Extracts

**DOI:** 10.1021/acsomega.6c00723

**Published:** 2026-05-07

**Authors:** Fernanda Gonçalves de Oliveira, Tatiana de Muros Amaral Barcellos, Talita Luisa Ribeiro Weberling, Thiago Silveira Alvares, Rodrigo de Siqueira Melo

**Affiliations:** † Nutrition and Exercise Metabolism Research Group, Food and Nutrition Institute, Multidisciplinary Center UFRJ-Macaé, Federal University of Rio de Janeiro, Avenida Aluízio da Silva Gomes, 50, Bairro da Glória, CEP: 27930-560 Macaé, Rio de Janeiro, Brazil; ‡ Electroanalytics and Advanced Materials Group, Multidisciplinary Institute of Chemistry, Multidisciplinary Center UFRJ-Macaé, Federal University of Rio de Janeiro, Avenida Aluízio da Silva Gomes, 50, Bairro da Glória, CEP: 27930-560 Macaé, Rio de Janeiro, Brazil

## Abstract

The increasing demand for rapid and reliable methods to characterize the electrochemical behavior of plant-derived bioactive materials has motivated the exploration of alternative electroanalytical approaches. In this study Electrochemical Impedance Spectroscopy (EIS) is employed as the primary analytical tool to characterize interfacial properties of carbon paste electrodes (CP) modified with beetroot (BET) and spinach (SPI) extracts. Impedance parameters were extracted using equivalent circuit modeling and correlated with the antioxidant composition of the extracts. Modification with beetroot extract led to a pronounced decrease in the primary charge-transfer resistance from 6.94 × 10^5^ Ω (CP) to 2.72 × 10^4^ Ω (BET), indicating facilitated interfacial electron-transfer processes associated with betalain-rich layers. In contrast, the spinach-modified electrode exhibited a higher charge-transfer resistance (9.90 × 10^6^ Ω) and a diffusion-controlled impedance response, consistent with the formation of a more resistive interfacial film. The results demonstrate that EIS provides distinct and reproducible impedance signatures for extract-modified electrodes, reflecting differences in interfacial charge-transfer kinetics, capacitive behavior, and mass-transport limitations. This work highlights the usefulness of EIS for characterizing the electrochemical interfacial behavior of electrodes modified with plant-derived extracts.

## Graphical abstract

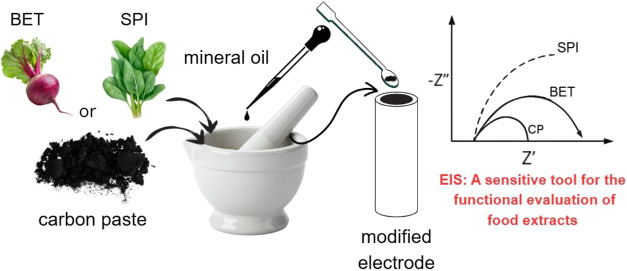

## 1 Introduction

The growing interest in bioactive compounds found in plants has driven the search for innovative analytical methodologies capable of characterizing their physicochemical and electrochemical properties in a rapid, sensitive, and sustainable manner.^1^ Natural extracts obtained from foods rich in pigments and phenolic compounds, such as beetroot (*Beta vulgaris*) and spinach (*Spinacia oleracea*), have attracted considerable attention owing to their high antioxidant capacity and the presence of redox-active molecules, including betalains, flavonoids, chlorophylls, and phenolic acids.^2^ These compounds play a key role in neutralizing reactive oxygen species (ROS), thereby contributing to the prevention of oxidative processes in both biological and food systems.^3^

Although traditional spectrophotometric methods such as 2,2-diphenyl-1-picrylhydrazyl (DPPH), 2,2-azino-bis­(3-ethylbenzothiazoline-6-sulfonic acid) (ABTS), and ferric reducing antioxidant power (FRAP) are widely employed to assess the antioxidant activity of natural extracts, these techniques present limitations with respect to reproducibility, analysis time, and reagent specificity.^4^ In this context, electroanalytical methods have emerged as powerful tools for the evaluation of redox-related processes in complex systems, offering higher sensitivity, faster analysis, and reduced waste generation.^5^ Rather than directly reporting thermodynamic redox potentials, parameters derived from techniques such as electrochemical impedance spectroscopy (EIS) provide insight into interfacial charge-transfer kinetics and surface physicochemical properties.

However, despite recent advances in the development of electrochemical sensors and methodologies for antioxidant evaluation, benchmark reviews—such as that published by Patil et al.^3^—do not include EIS among the techniques discussed. Most studies have focused on voltammetric and amperometric approaches, revealing a significant gap in the exploration of EIS as a tool for characterizing the electrochemical interfacial behavior of plant-derived matrices. Given that EIS provides unique insights into charge-transfer resistance, double layer capacitance, and interfacial electron-transport mechanisms, its application can offer a more comprehensive understanding of the redox behavior of bioactive compounds.^6^ Therefore, employing EIS in the functional assessment of natural extracts represents a promising yet underexplored avenue.

Among the available electrochemical techniques, EIS stands out as a nondestructive, highly sensitive method capable of describing interfacial phenomena in detail. From the analysis of Nyquist and Bode plots, physicochemical parameters that reflect the electrochemical behavior of natural materials—such as charge-transfer resistance (*R*_ct_) and double-layer capacitance—can be extracted. In this study, the term *R*_ct_ refers exclusively to the resistance associated with the primary interfacial electron-transfer process at the electrode surface. Any additional resistive contributions related to film formation, ion transport, or other interfacial phenomena are treated separately and are not included in the *R*_ct_ values unless explicitly stated. These impedance-derived parameters provide insight into interfacial charge-transfer kinetics and surface physicochemical properties, rather than directly reflecting thermodynamic redox potential.^7^ Consequently, EIS has gained increasing prominence in studies involving functional materials and biosensing interfaces. However, its application to plant-derived extracts remains insufficiently systematized. There is a lack of studies establishing EIS-derived descriptors that can be quantitatively correlated with antioxidant composition, as well as a lack of standardized electrochemical workflows capable of distinguishing between film-induced resistive effects and genuine redox-mediated charge-transfer processes in complex plant matrices.

The incorporation of plant extracts into carbon paste electrodes represents an innovative strategy for investigating the redox behavior of natural compounds. Pigments and antioxidants present in these extracts may influence interfacial electron-transfer processes, thereby modifying impedance responses. Recent studies have shown that electrode modification with plant extracts can alter charge-transfer resistance and capacitive behavior, suggesting a relationship between chemical composition and electrochemical performance.^8^

This study aims to investigate the electrochemical behavior of carbon paste electrodes modified with beetroot and spinach extracts, using EIS as the primary analytical tool. Specifically, we evaluate whether impedance-derived parameters can serve as quantitative descriptors of interfacial behavior associated with antioxidant-rich extracts and explore their correlation with electrochemically estimated antioxidant capacity. By addressing the current lack of systematic EIS-based characterization of plant matrices, this work contributes to advancing electrochemical strategies for the functional evaluation of natural products and bioactive compounds.

Unlike most electrochemical studies on antioxidants, which rely primarily on voltammetric peak currents or spectrophotometric radical-scavenging assays, the present work introduces Electrochemical Impedance Spectroscopy (EIS) as a quantitative tool to probe interfacial properties induced by plant-derived extracts. Rather than measuring antioxidant activity indirectly, EIS enables the discrimination between charge-transfer kinetics, capacitive behavior, and diffusion-controlled processes associated with extract-modified interfaces. This approach provides an electrochemical “fingerprint” of plant extracts that is not accessible through conventional voltammetric or optical techniques.

## 2 Results and Discussion

### 2.1 Study of Electrical Parameters from Equivalent Circuit Models

The impedance spectra obtained for unmodified CP and electrodes functionalized with BET and SPI extracts revealed substantial modifications in the interfacial structure and charge-transfer mechanisms. These effects were interpreted using fitted equivalent circuit models (Figure 1) and the extracted electrochemical parameters are summarized in Table 1. The modeling of EIS data using equivalent circuits (ECs) enables quantitative discrimination of the physicochemical processes occurring at the electrode–electrolyte interface and highlights the increase in interfacial complexity induced by surface modification. The solution resistance (*R*_1_) appears as the first series element in all circuits, with values ranging from 3.88 × 10^3^ Ω for CP to 3.69 × 10^4^ Ω for BET and 3.18 × 10^4^ Ω for SPI. These values indicate that the ohmic contribution of the electrolyte, although large in absolute terms (10^3^–10^4^ Ω), is small relative to the charge-transfer resistance (*R*_ct_) and therefore does not dominate the overall impedance response.

**Figure 1:**
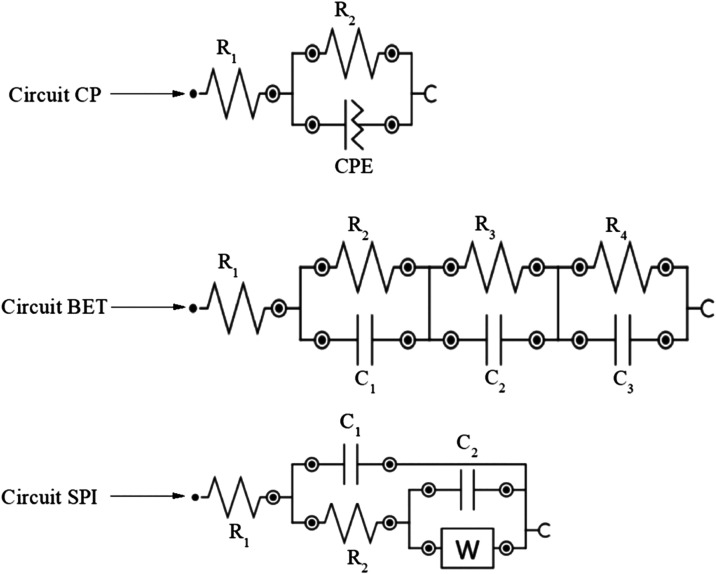
Proposed equivalent circuits used to fit the EIS data of the pure CP and BET- and SPI- modified electrodes. *R*_1_, solution resistance; *R*_2_ (*R*_ct_), primary charge-transfer resistance at the electrode/electrolyte interface; *R*_3_ (Rion), ion-transport resistance through the extract film; *R*_4_ (Rint), additional interfacial resistance; *C*_1_–*C*_3_, double-layer capacitances; CPE, constant phase element; *W*, Warburg diffusion element.

**Table 1:** Parameters of the Equivalent Circuits Obtained from Fitting the Experimental EIS Data for the CP and Those Modified with BET and SPI Extracts^a^

									CPE	
Sample	*R*_1_ (Ω)	*R*_2_ (Ω)	*R*_3_ (Ω)	*R*_4_ (Ω)	*C*_1_ (μF)	*C*_2_ (μF)	*C*_3_ (μF)	*W* (S s^1/2^)	*Y*_0_ (S s*^n^*)	*n*
CP	3.88 × 10^3^ (±0.65%)	6.94 × 10^5^ (±5.15%)	---	---	---	---	---	---	19.2 (±0.85%)	0.669 (±0.47%)
BET	3.69 × 10^4^ (±0.76%)	2.72 × 10^4^ (±4.78%)	2.65 × 10^5^ (±1.26%)	1.39 × 10^5^ (±4.24%)	0.346 (±5.51%)	0.349 (±1.65%)	0.006 (±6.89%)	---	---	---
SPI	3.18 × 10^4^ (±0.32%)	9.90 × 10^6^ (±0.64%)	---	---	4.75 × 10^–6^ (±1.07%)	0.068 (±2.10%)	---	3.06 × 10^–8^ (±2.47%)	---	---

a Values in parentheses indicate the standard deviation of the fitting.

#### 2.1.1 Unmodified CP Electrode

The unmodified CP electrode was adequately described by the equivalent circuit *R*_1_ + (*R*_2_||CPE), representing a single interfacial time constant associated with a dominant charge-transfer process at the electrode–electrolyte interface. In this model, the charge-transfer resistance *R*_2_ (6.94 × 10^5^ Ω) represents the main barrier to electron transfer, while the constant phase element (CPE) accounts for the nonideal capacitive behavior typically observed in heterogeneous carbon surfaces. The CPE exponent *n* (0.669) indicates significant surface heterogeneity and roughness, which is consistent with the porous microstructure commonly reported for carbon paste electrodes.^9^

Because the CP model includes a constant phase element (CPE) instead of an ideal capacitor, the effective double-layer capacitance (*C*_dl_) was estimated using the Brug relation, which allows conversion of the nonideal CPE response into an equivalent capacitance for depressed semicircles observed in Nyquist diagrams. Effective capacitance can be calculated according to

$$C_{dl}={\left[Y_{0}{\left(R_{s}^{-1}+R_{\mathrm{ct}}^{-1}\right)}^{n-1}\right]}^{1/n}$$

where *Y*_0_ and *n* correspond to the CPE parameters, *R*_s_ is the solution resistance, and *R*_ct_ is the charge-transfer resistance.

This approach enables the estimation of an equivalent double-layer capacitance for systems exhibiting nonideal capacitive behavior. The calculated *C*_dl_ value (0.224 μF, Table 2) reflects the intrinsic interfacial polarization of the bare carbon paste electrode. From the characteristic resistance and capacitance values, the interfacial time constant was estimated as τ ≈ 0.53 s, corresponding to a characteristic frequency *f*_max_ ≈ 0.30 Hz. This result is consistent with the phase-angle plot (Figure 4), in which the CP electrode exhibits a single-phase maximum in the low-frequency region, indicating a dominant interfacial relaxation process associated with charge transfer.

**Table 2:** Electrochemical Parameters Obtained from Electrochemical Impedance Spectroscopy (EIS) Analysis of the CP Electrode and Electrodes Modified with BET and SPI Extracts

Sample	*C*_dl_ (μF)	τ (s)	*f*_max_ (Hz)
CP	0.224	0.53	0.30
BET	0.701	0.026	6.0
SPI	4.45	5.30	0.03

#### 2.1.2 BET-Modified Electrode

Modification of the electrode with the BET extract significantly altered electrochemical behavior of the interface, resulting in a more complex equivalent circuit described by *R*_1_ + (*R*_2_||*C*_1_) + (*R*_3_||*C*_2_) + (*R*_4_||*C*_3_). The presence of three distributed RC subcircuits suggests the existence of multiple interfacial processes or electroactive domains on the surface of the modified electrode. Such behavior is frequently observed in systems containing adsorbed organic films or heterogeneous interfacial layers.^10^

The first element (*R*_2_||*C*_1_) (*R*_2_ = 2.72 × 10^4^ Ω; *C*_1_ = 0.346 μF) may be associated with the primary charge-transfer process near the carbon surface. The second element (*R*_3_||*C*_2_) (*R*_3_ = 2.65 × 10^5^ Ω; *C*_2_ = 0.349 μF) likely reflects additional interfacial polarization processes related to the presence of the adsorbed extract layer, whereas the third element (*R*_4_||*C*_3_) (*R*_4_ = 1.39 × 10^5^ Ω; *C*_3_ = 0.006 μF) may correspond to interfacial processes occurring at the boundary between the organic film and the electrolyte.

For this configuration, the overall double-layer capacitance was estimated as the sum of the individual capacitive contributions (*C*_dl_ = *C*_1_ + *C*_2_ + *C*_3_), since these elements represent spatially distributed interfacial regions contributing collectively to interfacial polarization. Using the fitted parameters (Table 1), the calculated value is *C*_dl_ = 0.701 μF. The resulting *C*_dl_ value (0.701 μF) is higher than that observed for the unmodified CP electrode, indicating an expansion of the effective electroactive surface area and enhanced charge-storage capability after surface modification, likely related to the incorporation of betalain-type compounds present in the extract.^11^ According to the phase-angle plot (Figure 4), the phase maximum for the BET-modified electrode occurs at approximately *f*_max_ ≈ 6 Hz, corresponding to a characteristic time constant of τ ≈ 0.026 s. This shift of the phase maximum toward higher frequencies suggests fast interfacial processes, associated with the increased electroactive surface area and the presence of multiple electrochemically active domains introduced by the extract layer.

#### 2.1.3 SPI-Modified Electrode

The electrode modified with SPI exhibited a distinct equivalent circuit described by *R*_1_ + (*C*_1_||(*R*_2_ + (*C*_2_||*W*))), in which the presence of the Warburg element (*W*) indicates a significant contribution from diffusion-controlled processes.^7^ The charge-transfer resistance *R*_2_ (9.90 × 10^6^ Ω) is considerably higher than that observed for the unmodified CP electrode, explaining the large semicircle observed in the Nyquist plot (Figure 2) and indicating that the spinach extract layer introduces a substantial barrier to electron transfer at the electrode–electrolyte interface. Barrier likely affects the kinetics of oxygen reduction and other interfacial redox reactions occurring at the modified surface, thereby increasing the apparent charge-transfer resistance.

**Figure 2:**
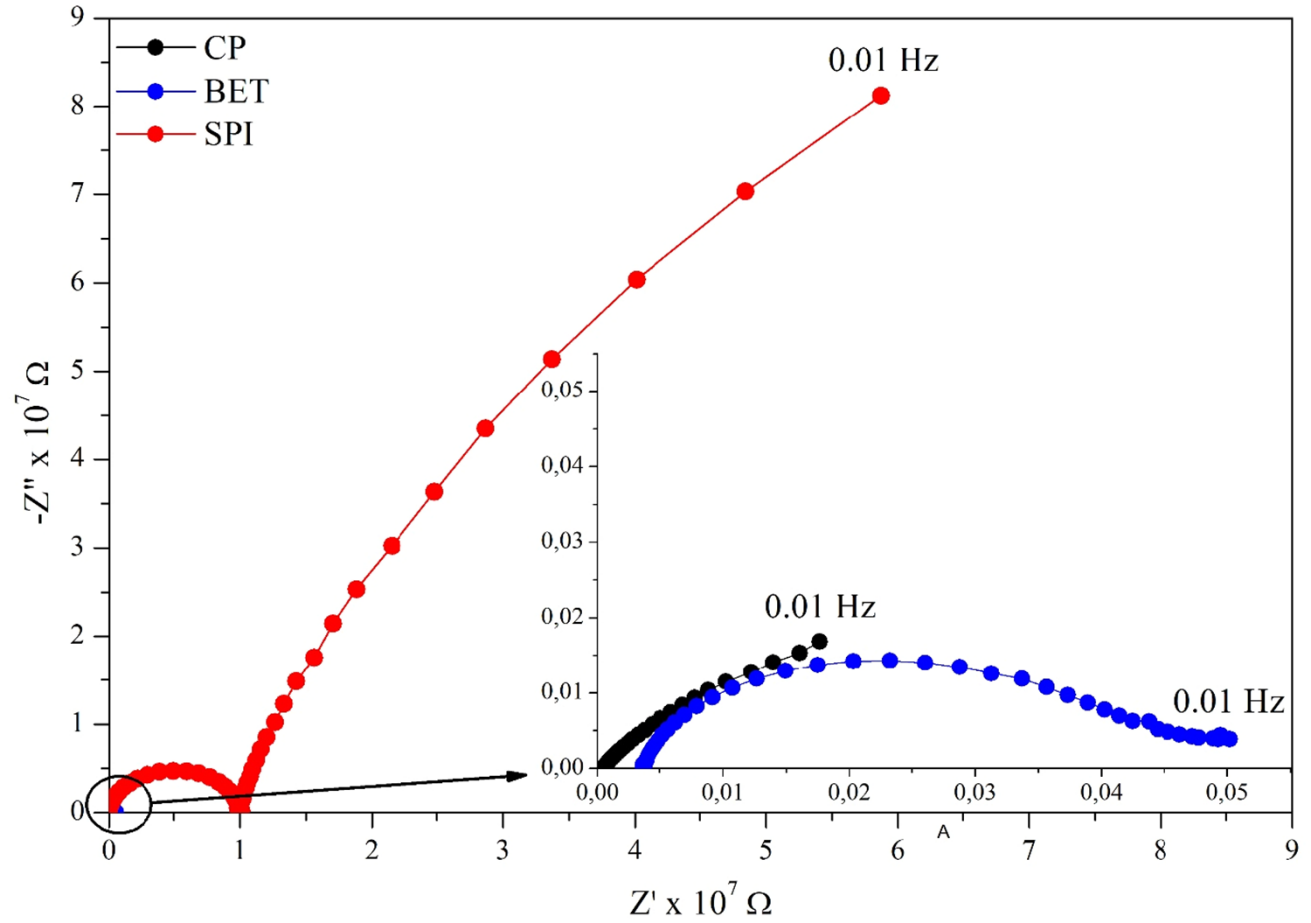
Nyquist diagrams for the CP electrode and electrodes modified with BET and SPI extracts. The inset shows a magnified view of the CP and BET electrodes.

The Warburg parameter (3.06 × 10^–8^ S s^1/2^) confirms the presence of diffusion limitations. Under the experimental conditions employed (PBS without an added redox couple), this diffusional contribution is associated with the mass transport of dissolved oxygen and other electroactive species present in the electrolyte toward the electrode surface, which participate in interfacial redox processes. This behavior suggests that the SPI-derived film is compact or less porous, thereby hindering ion transport toward the electrode surface.

The capacitance *C*_1_ (4.75 μF) is is consistent with the calculated *C*_dl_ value (4.45 μF) (Table 2), indicating that although the SPI layer increases interfacial resistance, it does not suppress interfacial polarization. Instead, the extract forms a resistive yet polarizable interfacial film. According to the phase-angle diagram, the phase maximum for the SPI-modified electrode occurs at approximately *f*_max_ ≈ 0.03 Hz, corresponding to a characteristic time constant of τ ≈ 5.3 s. This shift toward much lower frequencies indicates slower interfacial processes, consistent with the high charge-transfer resistance and the mass-transport limitations imposed by the organic layer formed on the electrode surface.

For the SPI configuration, the characteristic time constant was calculated using the dominant charge-transfer subcircuit

$$\tau =R_{2}C_{2}$$

and the corresponding characteristic frequency was determined as

$$f_{\max}=\frac{1}{2\pi R_{2}C_{2}}$$

The Warburg element was not included in the time constant calculation because it represents a frequency-dependent diffusion process rather than a discrete relaxation time. This approach allows the characteristic interfacial dynamics associated with the charge-transfer process to be isolated from the diffusional contribution present in the equivalent circuit.

Overall, the comparative EIS analysis reveals that the natural extracts significantly modify the interfacial dynamics of the carbon paste electrode. While the CP electrode exhibits simple behavior dominated by a single charge-transfer process, modification with BET promotes a substantial increase in interfacial capacitance and the formation of multiple electroactive domains, indicating a more heterogeneous and electrochemically active surface. In contrast, the SPI extract tends to form a more compact and resistive interfacial film, resulting in higher charge-transfer resistance and the appearance of diffusion-related impedance. These structural and electrochemical differences are clearly reflected in the Nyquist plots, phase-angle diagrams, and the parameters obtained from the fitted equivalent circuits.

#### 2.1.4 Quality of Fitting and Validation

Simpler equivalent circuit models were initially evaluated for all electrodes; however, they resulted in systematic deviations in the residuals and physically unrealistic parameter values. The adoption of multi-RC models was therefore necessary to accurately represent the distributed interfacial processes introduced by the extract-derived layers. The selection of the final models was guided by a combination of statistical goodness-of-fit, residual analysis, and physicochemical consistency of the fitted elements.

Fitting was performed using complex nonlinear least-squares (CNLS) with modulus weighting (|*Z*|^–2^). The χ^2^ values (10^–7^–10^–8^ range, Table 3) indicate excellent agreement between experimental and simulated spectra. In addition to χ^2^ values, model validity was assessed through residual distribution analysis and Kramers–Kronig consistency tests, ensuring linearity, causality, and stability of the impedance data.

**Table 3:** Fitted Equivalent Circuit Elements and Kramers–Kronig (χ^2^) Consistency Test Values Obtained for CP and Modified Electrodes with BET and SPI Extracts

		Kramers–Kronig (χ^2^)		
Sample	Circuit	*Z*	*Z*′	–*Z*″
CP	*R*_1_ + (*R*_2_||CPE)	7.04 × 10^–7^	3.96 × 10^–7^	3.08 × 10^–7^
BET	*R*_1_ + (*R*_2_||*C*_1_) + (*R*_3_||*C*_2_) + (*R*_4_||*C*_3_)	1.07 × 10^–8^	4.69 × 10^–8^	6.00 × 10^–8^
SPI	*R*_1_ + (*C*_1_||(*R*_2_ + (*C*_2_||*W*)))	2.98 × 10^–7^	1.93 × 10^–7^	1.05 × 10^–7^

The low relative standard deviations (<7%) indicate good reproducibility of the electrode preparation and fitting procedure. Kramers–Kronig validation was performed using the linear transform implemented in the fitting software, based on a distribution-of-relaxation-times (DRT) approach. Acceptance criteria included χ^2^ values below 10^–6^ and absence of systematic deviations between experimental and reconstructed spectra in both real and imaginary components. These results confirm the internal consistency of the impedance data and support the reliability of electrochemical interpretation.

### 2.2 Nyquist Plot Analysis

The Nyquist diagram (Figure 2), which plots the imaginary part of the impedance (−*Z*″) versus the real part (*Z*′), provides a direct visualization of the kinetic and mass-transport processes occurring at the electrode/electrolyte interface. The analysis begins with the high frequency intercept of the curve with the *Z*′ axis, which corresponds to the solution resistance (*R*_1_). For the three electrodes (CP, BET, and SPI), this intercept occurs at low values when compared to the total impedance of the system, although it is not negligible. According to Table 1, *R*_1_ values range from 3.88 × 10^3^ Ω (CP) to 3.69 × 10^4^ Ω (BET) and 3.18 × 10^4^ Ω (SPI), indicating that the ohmic contribution of the electrolyte is similar in magnitude among the samples and does not dominate the overall impedance response.

The charge-transfer resistance (*R*_ct_) is associated with the diameter of the semicircle observed in the medium and high-frequency region. In the present system, measured in PBS without the addition of an external redox probe, this resistance is attributed to interfacial electron-transfer processes involving dissolved oxygen and redox-active surface groups originating from the carbon paste and the plant extracts. Based on the fitted *R*_2_ values (Table 1), the correct order of magnitude for *R*_ct_ is *R*_ct_ (SPI) ≫ *R*_ct_ (CP) ≫ *R*_ct_ (BET). Numerically, the SPI-modified electrode exhibits the highest charge-transfer resistance (9.90 × 10^6^ Ω), followed by the unmodified CP electrode (6.94 × 10^5^ Ω), while the BET-modified electrode presents the lowest value (2.72 × 10^4^ Ω). The exceptionally large semicircle observed for SPI reflects a pronounced kinetic barrier to electron transfer, which can be attributed to the formation of a more resistive interfacial layer. Furthermore, in the low-frequency region, the SPI spectrum exhibits a straight line with an inclination of approximately 45°, characteristic of Warburg-type impedance. This behavior confirms that the electrochemical response of the SPI electrode is partially controlled by mass diffusion of electroactive species through the modifying film. This interpretation is fully consistent with the inclusion of the Warburg element (*W*) in the proposed equivalent circuit, *R*_1_ + (*C*_1_||(*R*_2_ + (*C*_2_||*W*))) (Figure 1).^12^

For the comparison between the CP and BET electrodes, the inset of Figure 2, which magnifies the high and medium-frequency region, is particularly informative. The CP electrode exhibits a semicircle significantly larger than that of BET, indicating a higher charge-transfer resistance. In contrast, the BET-modified electrode displays the smallest arc among the three systems, confirming the marked reduction in Rct promoted by the incorporation of the beetroot extract. This reduction suggests that the BET modification facilitates interfacial electron-transfer kinetics, due to improved surface conductivity and/or increased electroactive area. Therefore, rather than imposing an additional barrier, the extract layer promotes charge-transfer processes, in agreement with the fitted electrochemical parameters.^9^

### 2.3 Bode Diagram Analysis

The Bode Diagrams (Figure 3) are an indispensable analytical tool in EIS, providing essential complementary information about the dependence of impedance and phase angle on frequency. This representation allows for a more in-depth analysis and a clear distinction between the kinetic and mass transport processes occurring at the electrode/electrolyte interface.^13^ The impedance modulus plot (Figure 3) serves as a direct validation of the observations obtained from the Nyquist Diagram. The SPI electrode stands out by exhibiting the highest impedance modulus across the entire frequency spectrum, reaching close to 1.0 × 10^7^ Ω at 10^–2^ Hz. This result unequivocally reiterates that SPI is the electrode that presents the highest total system impedance, an indication of greater overall resistance to charge flow. In contrast, the CP and BET electrodes show significantly lower impedance moduli. A more detailed analysis, facilitated by the inset of Figure 3, reveals that in the low-frequency region the BET electrode exhibits a higher impedance modulus than the CP electrode. This difference is fully consistent with the decrease in *R*_ct_ previously observed in the Nyquist plot. It is important to note that, at the highest measured frequencies (∼10 kHz), the impedance modulus of all electrodes converges to a single value, which corresponds to the ohmic resistance of the solution (*R*_1_), confirming the low ohmic resistance of the electrolyte.^14^

**Figure 3:**
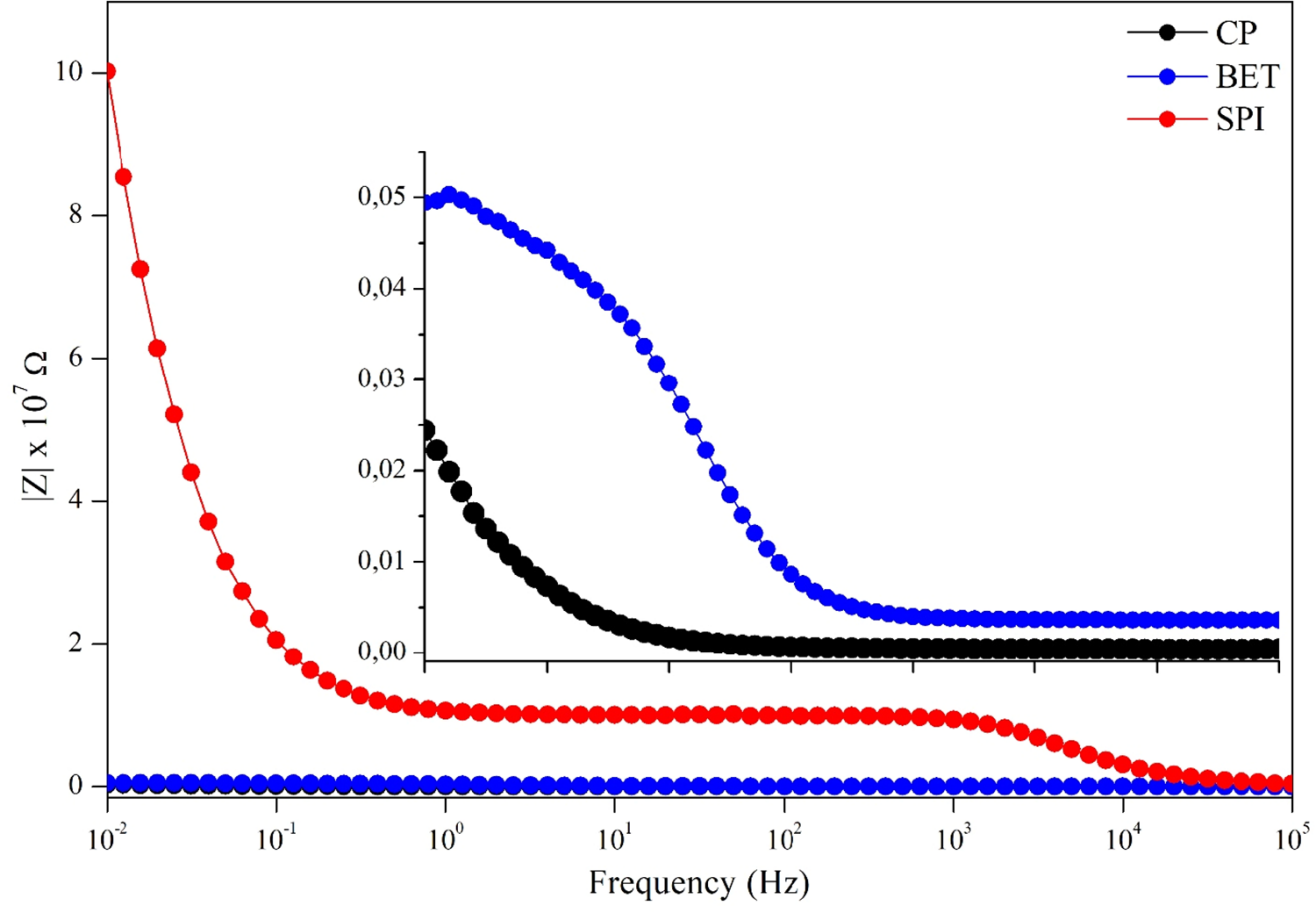
Bode diagrams (impedance) for the CP electrode and electrodes modified with BET and SPI extracts. The inset shows a magnified view of the CP and BET electrodes.

The phase angle (θ) plot (Figure 4) is fundamental for identifying the time constants (τ) and for characterizing the nature of the electrochemical processes, such as distinguishing between capacitive and resistive behavior.^15^ The SPI electrode presents a phase angle that approaching −85° at low frequencies (10^–2^ Hz). A phase angle close to −90° is the indicative of predominantly capacitive behavior, typically associated with a blocking interface or ideal capacitor-like response. Therefore, this behavior should not be interpreted as evidence of a Warburg diffusion region, which would instead be characterized by a phase angle close to −45°.^16^

**Figure 4:**
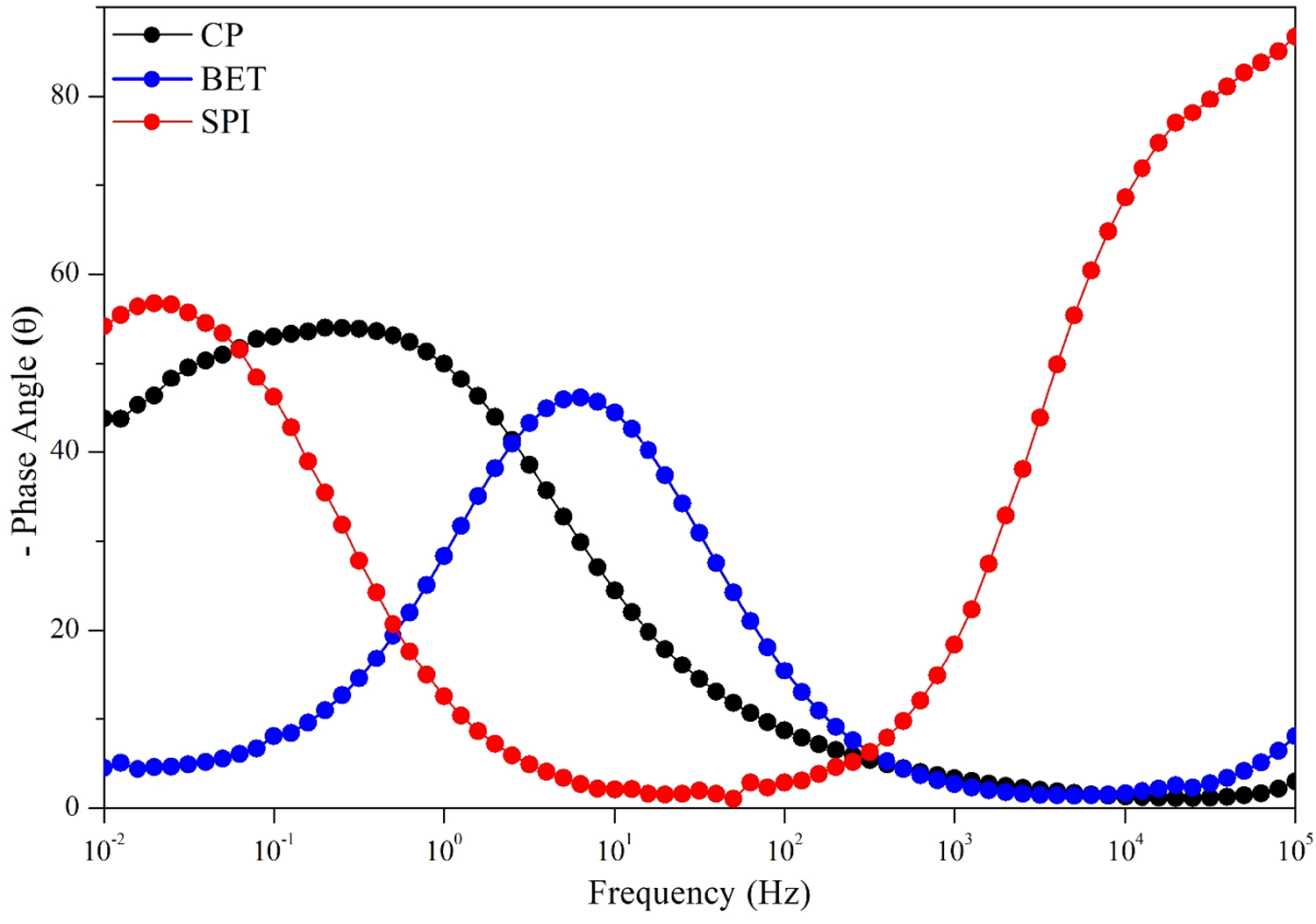
Bode diagrams (phase angle) for the CP electrode and electrodes modified with BET and SPI extracts. The inset shows a magnified view of the CP and BET electrodes.

The peak frequency (*f*_max_) for SPI is lower than BET electrode (Table 2). However, in systems described by complex equivalent circuits that include diffusion elements, *f*_max_ alone cannot be directly interpreted as evidence of intrinsically fast charge-transfer kinetics. Instead, it reflects the combined influence of the different processes contributing to the overall impedance. The BET electrode exhibits a phase angle peak near −45° at a frequency of approximately 0.2 Hz. The peak frequency (*f*_max_ ≈ 6 Hz) is related to the time constant by τ = 1/(2π*f*_max_), which corresponds to τ ≈ 0.026 s, consistent with the value reported in Table 2. A maximum phase angle close to −45° is characteristic of a mixed process in which both charge transfer and mass transport contribute significantly to the impedance. Furthermore, the width of the peak suggests the presence of multiple time constants, a complexity that is well represented by the more elaborate equivalent circuit *R*_1_ + (*R*_2_||*C*_1_) + (*R*_3_||*C*_2_) + (*R*_4_||*C*_3_) (Figure 1).^10^ The SPI electrode presents a phase-angle peak at approximately 0.03 Hz, corresponding to a time constant of τ ≈ 5.3 s according to the relation τ = 1/(2π*f*_max_), which is consistent with the value reported in Table 2. Finally, the CP electrode exhibits a phase-angle peak at 0.30 Hz, corresponding to τ ≈ 0.53 s. The fact that the maximum phase angle does not reach −90° indicates nonideal double-layer behavior, often modeled by a Constant Phase Element (CPE), suggesting that charge transfer plays a significant role in the overall impedance response.^17^

### 2.4 Electrochemical Implications and Antioxidant Capacity

The modification of carbon paste electrodes with plant extracts of beetroot and spinach produced significant changes in the electrochemical behavior of the interfaces. Electrochemical impedance spectroscopy analyses revealed that the bioactive compounds present in these extracts—mainly antioxidants such as betalains, polyphenols, and chlorophylls^22,23^ —may contribute to the modulation of the interfacial electrochemical properties between the electrode and the electrolyte. Although these extracts are widely recognized for their antioxidant properties, the electrochemical impedance measurements performed in this work do not directly probe radical scavenging reactions. Instead, the impedance response reflects interfacial electron-transfer and mass-transport processes associated with redox-active compounds present in the plant extracts adsorbed on the electrode surface.^17^ Therefore, the EIS responses reflect both the electroactive nature of the compounds and the morphology and conductivity of the layers formed on the carbon surface. It should be noted that both extracts contain maltodextrin as a carrier agent, which may influence the interfacial electrochemical response. Since a dedicated control experiment was not systematically evaluated, the contribution of maltodextrin cannot be fully separated from that of the plant-derived compounds and should be considered when interpreting the results.

#### 2.4.1 Behavior of the Beetroot-Modified Electrode

The beetroot extract, rich in betalains (betacyanins and betaxanthins) and phenolic compounds,^11,18^ exhibited facilitated interfacial electron-transfer behavior. As shown in Figure 2, the CP + BET electrode presented a marked reduction in *R*_ct_ compared to the unmodified CP electrode, indicating that the presence of betalain compounds modifies the interfacial electron-transfer properties of the electrode surface. In the present system, measured in PBS without the addition of an external redox probe, the faradaic contribution during EIS may be associated with dissolved oxygen present in the aerated electrolyte, as well as with redox-active functional groups originating from the carbon paste matrix and the plant-derived compounds.

Betalains contain electroactive functional groups that may participate in these interfacial redox processes.^11^ The decrease in *R*_ct_ therefore suggests that the BET-modified surface suggests improved interfacial electron-transfer kinetics associated with these reactions, particularly those involving dissolved oxygen at the electrode interface. Upon adsorption onto the carbon surface, these molecules form a porous and functionalized structure, as evidenced by the increase in double-layer capacitance (*C*_dl_ = 0.701 μF). This behavior suggests an expansion of the effective interfacial area and modification of charge-storage properties. However, since no specific analyte was evaluated in the present study, this increase in capacitance should be interpreted as an intrinsic interfacial property of the modified electrode rather than direct evidence of analytical performance.^19^

Modeling of the EIS data using the equivalent circuit *R*_1_ + (*R*_2_||*C*_1_) + (*R*_3_||*C*_2_) + (*R*_4_||*C*_3_) revealed multiple response domains associated with the multilayered nature of the interface (Figure 4). The phase peak around −45° further supports the presence of distinct charge-transfer processes occurring across different regions of the betalain layer.^20^ Despite the reduction in *R*_ct_, a slight increase in total impedance was observed, which may be attributed to the formation of an adsorbed organic layer derived from betalain compounds on the electrode surface. This layer, while providing surface functionalization, may introduce an additional barrier to direct electron transfer and modify interfacial transport processes.

#### 2.4.2 Behavior of the Spinach-Modified Electrode

The spinach extract is a rich source of polyphenols, flavonoids, carotenoids, and chlorophylls,^21,22^ all known for their antioxidant properties and potential electroactivity. However, the CP + SPI electrode displayed a markedly different behavior compared to CP + BET. As shown in Figures 2 and 3, the system exhibited the highest total impedance and a strong diffusion-controlled response, evidenced by the Warburg region and the phase angle approaching −90°.

The low double-layer capacitance (*C*_dl_ = 4.45 × 10^–6^ μF) suggests that the compounds in the spinach extract formed a denser or less conductive layer that blocked part of the electrode surface accessible for double-layer formation. Although polyphenols are electroactive, the structural organization of this modifier layer appears to restrict ion and charge transport, making diffusion the rate-determining step of the electrochemical process.

The increase in Rct and the prominent diffusion limitation (Warburg element) indicate that the film behaves partially as a passivating barrier. Although this reduces electrocatalytic performance, such characteristics may be advantageous for selectivity, as mass-transport limitations can differentiate species of higher molecular weight or lower mobility.

#### 2.4.3 Comparative Analysis and Implications for Electrochemical Sensing

Comparative analysis of the EIS parameters demonstrates that beetroot and spinach extracts produce distinct electrochemical interfaces. The CP + BET electrode exhibits lower charge-transfer resistance and higher double-layer capacitance, indicating faster interfacial electron-transfer kinetics. These properties may be relevant for potential analytical sensing applications, although analytical sensitivity was not evaluated in the present study. The SPI-modified electrode presents higher resistance and diffusion-controlled behavior, which may influence mass transport at the interface. Such characteristics could be relevant for analytical selectivity in systems where diffusion plays a role, although this hypothesis requires validation with specific analytes. Overall, these results highlight how plant-derived extracts can modulate interfacial electrochemical properties of carbon paste electrodes, providing a basis for future studies exploring their electrochemical and potential analytical applications.

## 3 Conclusions

This study demonstrates that electrochemical impedance spectroscopy can be effectively applied as a primary analytical tool to characterize interfacial electrochemical properties of carbon paste electrodes modified with plant-derived extracts. The incorporation of beetroot and spinach extracts produced markedly different impedance responses, reflecting distinct interfacial structures and charge-transfer dynamics. The beetroot-modified electrode exhibited a lower primary charge-transfer resistance and increased double-layer capacitance, indicating facilitated interfacial electron-transfer behavior associated with betalain-rich layers. In contrast, the spinach-modified electrode showed a higher charge-transfer resistance and a pronounced diffusion-controlled response, consistent with the formation of a more compact and resistive interfacial film.

By decoupling charge-transfer resistance, capacitive effects, and diffusion-related contributions through equivalent circuit analysis, EIS enabled the identification of characteristic impedance signatures for each extract-modified interface. Importantly, the impedance responses observed in this work reflect interfacial electron-transfer and mass-transport processes associated with redox-active constituents, rather than direct measurements of radical-scavenging activity.

The novelty of this work lies in the use of EIS not as a complementary technique, but as a central analytical strategy to probe the functional electrochemical behavior of complex plant matrices. This approach provides a mechanistic electrochemical perspective that extends beyond conventional antioxidant assays and voltammetric methods. From an application standpoint, the ability to discriminate between electrocatalytically active and diffusion-limited interfaces suggests potential use of EIS for electrochemical screening of plant extracts, functional foods, and bioactive ingredients. Future studies may extend this methodology toward targeted analytical sensing and broader correlations with biological functionality.

## 4 Experimental Section

### 4.1 Beetroot and Spinach Extract Preparation and Characterization

Fresh beetroot and spinach were purchased from a local supermarket and processed separately. The beetroots were washed, peeled, and centrifuged using a food-grade centrifuge. The resulting juice was filtered and subsequently subjected to a second filtration step to remove remaining suspended solids. Maltodextrin (15%, w/w) was then added as a carrier agent, and the mixture was homogenized under magnetic stirring until complete dissolution. The homogenized solution was dried by spray drying using a Büchi B-290 mini spray dryer (Flawil, Switzerland) under standard operating conditions. Although the exact parameters were not recorded, the procedure followed typical conditions comparable to those applied for the spinach extract preparation. For the spinach extract, both leaves and stems were used in equal proportions (50 g each). A total of 100 g of fresh spinach was blended with 200 mL of distilled water to obtain a homogeneous juice. Maltodextrin (15%, w/w) was then added as a carrier agent to facilitate drying. The mixture was subsequently dried using a mini spray dryer (Büchi B-290, Flawil, Switzerland) under the following operating conditions: inlet temperature of 160 °C, feed rate of 30%, and airflow rate of 80%. Although the preparation procedures involved different initial matrices (pure beetroot juice and diluted spinach homogenate), and the exact spray-drying parameters were not identical or fully recorded, both extracts were converted into spray-dried powders using maltodextrin as a carrier agent. Therefore, differences in processing conditions may have contributed to the observed electrochemical behavior and should be considered when comparing the two systems.

The antioxidant capacity of the resulting beetroot and spinach extracts was estimated electrochemically using square wave voltammetry (SWV) with gallic acid as the reference standard. Measurements were performed using a three-electrode system consisting of a carbon paste working electrode, an Ag/AgCl reference electrode, and a platinum auxiliary electrode connected to a potentiostat. The measurements were carried out in phosphate-buffered saline (PBS, pH 7.4). The SWV parameters were: frequency of 100 Hz, amplitude of 40 mV, potential step of 5 mV, and potential window from 0.50 to 1.0 V. All measurements were performed in triplicate. Calibration curves were constructed using gallic acid as the reference antioxidant compound. The antioxidant content was expressed as milligrams of gallic acid equivalents per gram of spray-dried extract powder (mg GAE·g^–1^), which includes the maltodextrin carrier used during the spray-drying process. The values obtained were 3.06 ± 0.13 mg GAE·g^–1^ for the beetroot extract and 0.23 ± 0.06 mg GAE·g^–1^ for the spinach extract. These values represent an electrochemically derived estimate of antioxidant capacity and are provided only as a compositional reference for the materials used in electrochemical experiments. It should be noted that this parameter does not represent an independent chemical compositional analysis of the extracts, since it was obtained electrochemically and is expressed as gallic acid equivalents per gram of spray-dried powder containing maltodextrin. The antioxidant contents obtained in this study are consistent with values reported in the literature for spray-dried beetroot and spinach extracts, taking into account differences in extraction procedures, drying methods, and the presence of carrier agents such as maltodextrin. Beetroot extracts are commonly reported to exhibit higher antioxidant capacity than spinach-derived products, primarily due to their high betalain content, whereas spinach extracts typically present lower gallic acid equivalent values when diluted or processed with carrier matrices. Therefore, the values reported here are representative of processed plant extracts rather than fresh raw materials.

### 4.2 Preparation of the CP

The carbon paste (CP) was prepared by thoroughly mixing 0.1 g of graphite powder (synthetic graphite, particle size <50 μm, Sigma-Aldrich) with 50 μL of mineral oil (analytical grade, Sigma-Aldrich) in an agate mortar using a pestle for approximately 5 min until a homogeneous paste was obtained. Approximately 30 mg of this paste were then packed into the cavity of the electrode body (2.0 mm in diameter and 2.0 mm in depth), corresponding to a geometric working electrode area of 0.031 cm^2^. All impedance parameters reported correspond to the measured values without area normalization. Considering the cavity diameter (2.0 mm), the geometric area of the working electrode was 0.031 cm^2^. All impedance parameters reported correspond to the measured values without area normalization. Before each measurement, the electrode surface was renewed by gently polishing the paste on weighing paper to obtain a fresh and smooth surface. For reproducibility evaluation, independent electrodes were prepared for each replicate experiment.

The CPs modified with beetroot extract (BET) or spinach extract (SPI) were prepared following the same procedure, except that 0.01 g of the respective extract powder was added to the graphite–mineral oil mixture prior to homogenization. To evaluate the possible influence of the maltodextrin carrier used during spray drying, its contribution to the electrochemical response should be considered. Since a dedicated control experiment was not systematically evaluated in this study, the potential effect of maltodextrin on the impedance response cannot be completely excluded. It should be noted that maltodextrin was used as a carrier agent during the spray-drying process of both extracts, although at different proportions for beetroot and spinach preparations. Therefore, although the electrochemical responses observed for BET and SPI are likely influenced by the phytochemical composition of the extracts, a contribution from the maltodextrin carrier cannot be excluded and should be considered when interpreting the results. Before each electrochemical measurement, the electrode surface was renewed by gently polishing the paste on weighing paper to obtain a fresh and reproducible surface. Independent carbon paste electrodes were prepared for each replicate experiment to ensure experimental reproducibility.

### 4.3 Electrochemical Impedance Spectroscopy

Electrochemical impedance spectroscopy measurements were carried out using an Autolab 128N potentiostat/galvanostat at 25 ± 1 °C under laboratory ambient conditions. The working electrodes were CP, BET, and SPI, while a platinum disk (*d* = 2 mm) and an Ag/AgCl electrode served as the counter and reference electrodes, respectively. Although the counter electrode had a similar diameter to the working electrode cavity, its effective exposed area was larger than that of the carbon paste surface, minimizing counter-electrode polarization effects during the impedance measurements. All EIS measurements were performed in phosphate-buffered saline (PBS, 0.1 mol L^–1^, pH 7.4). Prior to impedance measurements, the electrodes were allowed to stabilize at the open circuit potential (OCP) for 300 s under naturally aerated conditions. Therefore, the faradaic processes contributing to the impedance response are primarily associated with the reduction of dissolved oxygen present in the electrolyte, as well as redox-active functional groups originating from the plant extracts and surface functionalities of the carbon paste electrode. In such systems, the charge-transfer resistance (*R*_ct_) represents the kinetic barrier associated with these interfacial electron-transfer processes, while the diffusion component described by the Warburg element reflects the mass transport of dissolved oxygen and other electroactive species toward the electrode surface. A sinusoidal perturbation of 10 mV (RMS amplitude) was applied, and the impedance spectra recorded potentiostatically at the open circuit potential inside a Faraday cage. The frequency range investigated extended from 10 kHz to 0.01 Hz.

The impedance data were analyzed and presented as Nyquist and Bode plots. The consistency of the experimental data was verified by applying the Kronig–Kramers (K–K) test to ensure linearity, causality, and stability of the system. Equivalent electrical circuit models were then proposed to fit the experimental data using Nova software (Metrohm Autolab), applying a complex nonlinear least-squares fitting procedure with modulus weighting. Parameter constraints were applied to ensure physically meaningful values. Each reported parameter corresponds to the mean ± standard deviation obtained from at least three independently prepared electrodes. All EIS measurements were performed using at least three independently prepared electrodes for each condition (CP, BET, and SPI). All impedance parameters reported correspond to mean values obtained from at least three independent electrodes, with the associated standard deviations provided in the tables.

## References

[1] Zhang Q., Yue Y., Li X., Zhang C., Guo Y., Wang Z., Li J. (2025). Advances in analytical techniques for bioactive compound quantification
in medicinal plants: Innovations, challenges, and pharmaceutical applications. Microchem. J..

[2] Kumorkiewicz-Jamro A., Górska R., Krok-Borkowicz M., Mielczarek P., Popenda L., Lystvan K., Pamuła E., Wybraniec S. (2023). Unveiling alternative oxidation pathways
and antioxidant
and cardioprotective potential of amaranthin-type betacyanins from
spinach-like atriplex hortensis var. ‘rubra. J. Agric. Food Chem..

[3] Patil N. D., Bains A., Sridhar K., Sharma M., Dhull S. B., Goksen G., Chawla P., Inbaraj B. S. (2025). Recent advances
in the analytical methods for quantitative determination of antioxidants
in food matrices. Food Chem..

[4] Vicente-Zurdo D., Gómez-Mejía E., Morante-Zarcero S., Rosales-Conrado N., Sierra I. (2025). Analytical strategies
for green extraction,
characterization, and bioactive evaluation of polyphenols, tocopherols,
carotenoids, and fatty acids in agri-food bio-residues. Molecules.

[5] Amorim I., Bento F. (2024). Electrochemical sensors based on
transition metal materials for phenolic
compound detection. Sensors.

[6] Randviir E. P., Banks C. E. (2022). A review of electrochemical
impedance spectroscopy
for bioanalytical sensors. Anal. Methods.

[7] Lazanas A. C., Prodromidis M. I. (2023). Electrochemical
impedance spectroscopy - a tutorial. ACS Meas.
Sci. Au.

[8] Berkel C., Özbek O. (2024). Green electrochemical
sensors, their applications andgreenness
metrics used: A review. Electroanalysis.

[9] Mesa R., Khan S., Sotomayor M. D. P. T., Picasso G. (2022). Using carbon paste
electrode modified with ion imprinted polymer and mwcnt for electrochemical
quantification of methylmercury in natural water samples. Biosensors.

[10] Zhang L., Dai D., Li C., Dang Y., Zheng R., Wang Z., Wang Y., Cui Y., Arandiyan H., Shao Z., Sun H., Zhuang Q., Liu Y. (2024). Recent advances
in electrochemical impedance spectroscopy for solid-state batteries. Energy Storage Mater..

[11] Meena J., Santhakumar K., Kumar S. A. (2023). Redox-active green
electrode: Plant-based
betanin immobilized on carbon black for drift-free voltammetric and
potentiometric ph sensor applications. ACS Omega.

[12] Bard, A. J. ; Faulkner, L. R. Electrochemical Methods: Fundamentals and Applications, 3rd ed.; Wiley, 2022; p 1104.

[13] Magar H. S., Hassan R. Y. A., Mulchandani A. (2021). Electrochemical
impedance spectroscopy
(eis): Principles, construction, and biosensing applications. Sensors.

[14] Das S., Banerjee A., Nandi U., Ghosh A. (2025). Critical review on
the analysis of electrochemical impedancespectroscopy data. J. Appl. Phys..

[15] Brett C. M. A. (2022). Electrochemical
impedance spectroscopy in the characterisation and application of
modified electrodes for electrochemical sensors and biosensors. Molecules.

[16] Shirsath A. V., Raël S., Bonnet C., Schiffer L., Bessler W., Lapicque F. (2020). Electrochemical pressure impedance
spectroscopy for
investigation of mass transfer in polymer electrolyte membrane fuel
cells. Curr. Opin. Electrochem..

[17] Çakar S., Özacar M. (2025). Electrochemical
sensors for the determination of polyphenols
as antioxidants from natural sources: A comprehensive review of sensor
development and characterization. Talanta Open.

[18] Duqueyroix A., Aider-Kaci F. A., Aider M. (2022). Impact of the anodic and cathodic
electro-activation treatment on the physico-chemical and antioxidant
capacity of red beetroot juice. ACS Omega.

[19] Moorthy M., Karnan M., Balaji S. S., Gokulnath S., Sathish M. (2022). Nanoarchitectonics with beetroot peel waste derived
activated carbon for improved electrochemical performances in supercapacitors
using redox additive electrolyte. J. Electroanal.
Chem..

[20] Munteanu I. G., Apetrei C. (2021). A review on electrochemical
sensors and biosensors
used in chlorogenic acid electroanalysis. Int.
J. Mol. Sci..

[21] de
A Pedrosa V., Codognoto L., Avaca L. A. (2003). Electroanalytical
determination of 4-nitrophenol by square wave voltammetry on diamond
electrodes. J. Braz. Chem. Soc..

[22] Pisoschi A. M., Pop A., Cimpeanu C., Predoi G. (2016). Antioxidant capacity determination
in plants and plant-derived products: A review. Oxid. Med. Cell. Longevity.

[23] Wybraniec S., Paweł S., Aneta S., Boris N., Zbigniew P., Tadeusz M. (2011). Antioxidant activity of betanidin: Electrochemical
study in aqueous media. J. Agric. Food Chem..

